# Selepressin and Arginine Vasopressin Do Not Display Cardiovascular Risk in Atherosclerotic Rabbit

**DOI:** 10.1371/journal.pone.0165422

**Published:** 2016-10-27

**Authors:** Olivier Boucheix, Robert Blakytny, Gerard Haroutunian, Marie Henriksson, Regent Laporte, Stephane Milano, Torsten M. Reinheimer

**Affiliations:** 1 Charles River France, Saint Germain sur l’Arbresle, France; 2 TransEdEng, Ulm, Germany; 3 Echographie & Cardiologie, Marseille, France; 4 Ferring Pharmaceuticals A/S, Copenhagen, Denmark; 5 Laporte & Associates LLC – Biotech & Pharma R&D Consultants, San Diego, California, United States of America; 6 Double Strand Consulting, Saint Nizier d'Azergues, France; Kurume University School of Medicine, JAPAN

## Abstract

**Background:**

Septic shock remains associated with significant mortality rates. Arginine vasopressin (AVP) and analogs with V_1A_ receptor agonist activity are increasingly used to treat fluid-resistant vasodilatory hypotension, including catecholamine-refractory septic shock. Clinical studies have been restricted to healthy volunteers and catecholamine-refractory septic shock patients excluding subjects with cardiac co-morbidities because of presumed safety issues. The novel selective V_1A_ receptor agonist selepressin, with short half-life, has been designed to avoid V_2_ receptor-related complications and long-term V_1A_ receptor activation. Cardiovascular safety of selepressin, AVP, and the septic shock standard of care norepinephrine was investigated in a rabbit model of early-stage atherosclerosis.

**Methods:**

Atherosclerosis was established in New Zealand White rabbits using a 1% cholesterol-containing diet. Selepressin, AVP, or norepinephrine was administered as cumulative intravenous infusion rates to atherosclerotic and non-atherosclerotic animals.

**Results:**

Selepressin and AVP induced a slight dose-dependent increase in arterial pressure (AP) associated with a moderate decrease in heart rate, no change in stroke volume, and a moderate decrease in aortic blood flow (ABF). In contrast, norepinephrine induced a marked dose-dependent increase in AP associated with a lesser decrease in the heart rate, an increase in stroke volume, and a moderate increase in ABF. For all three vasopressors, there was no difference in responses between atherosclerotic and non-atherosclerotic animals.

**Conclusion:**

Further studies should be considered using more advanced atherosclerosis models, including with septic shock, before considering septic shock clinical trials of patients with comorbidities. Here, selepressin and AVP treatments did not display relevant cardiovascular risk in early-stage rabbit atherosclerosis.

## Introduction

Septic shock remains associated with mortality rates of 20–50% in critical care medicine despite some improvements in recent years [1−4]. Shock is the result of a systemic inflammatory response syndrome (SIRS) inducing systemic vasodilatation and increased vascular permeability, both leading to hypotension that becomes unresponsive to fluid resuscitation. Administration of the vasopressor norepinephrine (NE) is septic shock standard of care, although this catecholamine is not registered for such a clinical use [5]. In many cases NE administration eventually fails to stabilize arterial pressure (AP), termed the catecholamine-refractory stage, which leads to multiple organ failure syndrome and mortality. The neuroendocrine hormone arginine vasopressin (AVP) and analogs with selective V_1A_ receptor-agonist activity are increasingly used to treat catecholamine-refractory septic shock and the irreversible phase of fluid-resuscitated hemorrhagic shock, another form of distributive shock like septic shock [6].

Both decreased and increased mortality rates have been reported in association with AVP treatment of these two forms of distributive shock. For example, a retrospective study of acute trauma patients in the irreversible phase of hemorrhagic shock showed that AVP administration was associated with increased mortality compared to patients receiving other vasopressors [7]. In contrast, in the Vasopressin and Septic Shock Trial, a placebo-controlled randomized clinical trial, AVP administration reduced mortality compared to NE, although this was only significant in patients with less severe condition [8]—there was no significant difference in patients with more severe septic shock and in the trial patients overall. Interestingly, in a subset of patients, who received corticosteroids, AVP administration also reduced mortality compared to NE [9]. Amongst the criticisms of the Vasopressin and Septic Shock Trial was its exclusion of patients with underlying heart disease; it was suspected that the inclusion of such patients, a situation more representative of the general population, may lead to additional safety issues related to AVP administration potentially culminating in increased mortality [10].

Induction of atherosclerosis in the rabbit using a high-cholesterol diet is a well-established animal model of heart disease [11]. We investigated in this model, the cardiovascular safety of the V_1A_ receptor agonist selepressin (FE 202158) and AVP in comparison with NE. Selepressin is a nonameric peptide analog of AVP highly selective for the V_1A_ receptor in contrast with the mixed agonism of AVP at the V_1A_, V_2_, and OT receptors [12–14]. While V_1A_ receptor activation induces vasoconstriction, maintaining AP in septic shock [15], V_2_ receptor activation may have deleterious effects in such a setting, including vasodilation and release of the pro-coagulant factor VIII and von Willebrand factor [16,17]. Septic shock is a pro-thrombotic condition often complicated by disseminated intravascular coagulation [18]. In addition, selepressin has a short half-life in healthy rats and dogs of approximately 20 min and 30 min, respectively (unpublished results), and bore no relevant coronary ischemic liability in healthy dogs [19]. Therefore, selepressin should avoid complications related to V_2_ receptor activation and long-term V_1A_ receptor activation. It was shown to be superior to AVP in animal models of severe sepsis and septic shock [20–22], has completed Phase IIa clinical trials for the treatment of vasodilatory hypotension in septic shock (NCT01000649; NCT01612676), and has entered combined phase II/III of clinical development (NCT02508649). Should administration of selepressin and AVP not pose any additional safety issues, including cardiovascular liability, in the rabbit model of early-stage atherosclerosis used here as a first stage, more advanced animal disease models should be considered to challenge the argument that patients with underlying heart disease should be excluded and support broadening of the inclusion criteria in further clinical trials of these vasopressors.

## Materials and Methods

### Materials

Selepressin as selepressin acetate (0510–103) and AVP (208044–01) were supplied by Ferring (Copenhagen, Denmark) and NE (T-2010B, 4201790 and T-2011B) was obtained from Aguettant (Lyon, France).

### Animals

Thirty male New Zealand White rabbits (2.6–3.6 kg, Centre d’élevage G. Achard de la Vente, St Mars d'Egrenne, France), aged 3–4 months were individually housed in stainless steel mesh cages (500 × 600 × 400 mm) at 19–25°C and ≥35% humidity with a 12-h light/dark cycle. Each animal received Bolapin (Huttepain Bouix, Le Mans, France) at approximately 60 g/day as standard diet (non-atherosclerotic animals) or supplemented with1% cholesterol for 6 weeks to induce atherosclerosis (both n = 15). The animals were fasted for at least 12 h before clinical laboratory blood sampling, on the day of the experiment, and before scheduled necropsy. Water was supplied *ad libitum*. On completion of cholesterol feeding, the animals were prepared surgically to determine cardiovascular variables and infused intravenously with selepressin, arginine vasopressin (AVP), or norepinephrine (NE) at incremental rates.

This study was conducted in compliance with Good Laboratory Practices, including retrospective formulation analysis and plasma sample bioanalysis to confirm appropriate exposure. All experimental procedures were approved by the Local Ethics Committee of WIL Research Europe Lyon, France, with the welfare agreement under the number 7.2009, and conformed to the guidelines from Directive 2010/63/EU.

### Surgical Preparation

Following overnight fasting, the rabbits were pre-anesthetized intramuscularly using a mixture of 15 mg/kg ketamine hydrochloride (Imalgène 500^®^, Rhône Mérieux, Lyon, France) and 0.7 mg/kg xylazine hydrochloride (Rompun^®^ 2%, KVP, Kiel, Germany). The animals were tracheotomized and maintained under isoflurane anesthesia (2% v/v isoflurane in oxygen [isoflurane: AErrane^®^, Baxter, Maurepas, France]). The electrocardiogram was monitored continuously (Lead II derivation) during surgery. A femoral vein was cannulated using a polyvinyl chloride catheter to administer selepressin, AVP, NE, or vehicle while a carotid artery was cannulated for blood sampling. A Millar catheter-tip transducer was introduced into the femoral artery to determine arterial pressure. The animals were infused intravenously using Ringer lactate (Lavoisier, Paris, France) at 2 mL/h.

### Doppler Ultrasonography

A left parasternal image window was used to obtain a long-axis view, visualizing the entire ascending aorta, the innominate artery branch, and the left common carotid arteries in the same plane. Prior to dosing, all of the animals (atherosclerotic [A] and non-atherosclerotic [NA]) were examined by 2-dimensional ultrasonography using a 15-MHz linear probe. The thoracic aorta was visualized at up to 30 frames/s using a 120-μm axial resolution. An adequate penetration depth was set to several centimeters beyond the far wall of the ascending aorta. Intima-media thickness (IMT) immediately proximal to the innominate artery branch site was determined in a consistent manner in each animal using the arterial branching points as anatomic landmarks.

Doppler ultrasonographic measurements were performed using Acuson Séquoia 526 color Doppler equipment (Siemens, Saint-Denis, France). Aortic flow measurements were performed from a trans-hepatic view for perfect alignment (cos(theta) of the Doppler equation ≈1). Time velocity integrals and aortic diameter were determined for each time point by aortic echography.

Aortic blood flow (ABF, mL/min) was calculated according to the following formula:

ABF (mL/min) = SV (mL/beat)×HR (beats/min)

where:

- HR: heart rate
- SV (stroke volume, mL) = ITV_Ao_ (cm) × S_Ao_ (cm^2^)
- ITV_Ao_: Integral time velocity of the aorta evaluated from the mean of three to five consecutive velocity waveforms (i.e., three to five heartbeats)
- S_Ao_ (Section of the aorta) = (π × D_Ao_^2^)/4
- D_Ao_: Aortic diameter (cm) determined by aortic echography close to the maximal velocity measurement site.

### Drug Administration

Before treatment, the animals (n = 5 for each drug and for A vs. NA) were infused with the corresponding vehicle (10 mM acetate buffer at 9% v/v in 0.9% NaCl for selepressin, 10 mM acetate buffer at 0.3% v/v in 0.9% NaCl for AVP, and in 0.9% NaCl for NE) at 1 mL.kg^−1^.h^−1^ for 15 min. Selepressin acetate, AVP, and NE were administered via the femoral vein. Specifically, seven or eight incremental infusion rates of selepressin (1, 3, 10, 30, 100, 300, 1000, and 3000 ng.kg^−1^.min^−1^), AVP (0.3, 1, 3, 10, 30, 100, 300, and 1000 ng.kg^−1^.min^−1^), or NE (10, 30, 100, 300, 1000, 3000, and 10,000 ng.kg^−1^.min^−1^) were each administered at 1 mL.kg^−1^.h^−1^ until the maximum hemodynamic effect was reached, or for a maximum of 15 min in cases when no hemodynamic change was observed.

### Drug Formulation Analysis and Plasma Bioanalysis

Blood for bioanalysis was sampled from all of the animals both before the first infusion (vehicle control) and for each infusion rate at the time of maximum hemodynamic effect. Quantification of selepressin, AVP, or NE in plasma samples was performed using validated liquid chromatography with tandem mass spectrometric detection for selepressin and AVP and validated liquid chromatography with colorimetric detection for NE. All of the animals treated with selepressin, AVP, or NE were exposed in a dose-dependent manner, with no difference between A and NA animals. The maximum selepressin plasma concentrations (mean ± standard deviation) were 329±52 and 367±48 ng/mL for A and NA animals, respectively, for AVP they were 122±27 and 100±46.2 ng/mL for A and NA, respectively, and for NE they were 301±126 and 345±164 ng/mL for A and NA, respectively.

### Cardiovascular Variables Collection and Analysis

AP and electrocardiogram signals were amplified (Hugo Sachs Elektronik, March-Hugstetten, Germany) and recorded continuously using a computerized data acquisition system (Notocord, Croissy-sur-Seine, France) with acquisition rates of 500 and 1000 Hz for arterial pressure and electrocardiogram, respectively.

### Lipid Variables

On completion of feeding for 6 weeks of a normal diet or a 1% cholesterol-containing diet, total serum cholesterol, triglycerides (TG), high-density lipoprotein-cholesterol (HDL-C), and low-density lipoprotein-cholesterol (LDL-C) concentrations were determined at the end of administration of the highest infusion rate tested for each vasopressor using a Beckman Coulter AU640 Chemistry autoanalyzer (Beckman Coulter, Villepinte, France).

### Biomarkers of Cardiac Injury

Serum aspartate aminotransferase and creatine kinase (CK) were determined at baseline (before administration of vasopressors) and at the end of administration of the highest infusion rate of selepressin, AVP, or NE using a Beckman Coulter AU640 Chemistry autoanalyzer. Serum cardiac troponin I was determined using a rabbit enzyme-linked immunosorbent assay kit (Life Diagnostics, West Chester, PA, USA).

### Histology

Animals were euthanized using an overdose of pentobarbital (Vetoquinol, 200 mg/mL) administered via the venous catheter. Three samples from the aorta of each animal were collected at necropsy and fixed in 10% neutral formalin. Tissue sampling and trimming were in accordance with the Registry of Industrial Toxicology Animal-data guide for rats. All sections were stained using hematoxylin and eosin.

### Data Collection and Statistical Evaluation

During baseline and for each infusion rate period of 15 min, arterial pressure and heart rate were continuously monitored and averaged over 30 s at every 3-minute interval. The integral time velocity of the aorta and aortic diameter were determined every 3 min in a similar manner.

The results were evaluated for each variable and each animal, and expressed as individual values and mean ± standard deviation. For each vasopressor (selepressin, AVP, or NE) of the A and NA animals, the following values were calculated:

Baseline: the mean value over the 15-minute period before administration of the lowest infusion rate of vasopressor (defined as the vehicle alone).

Maximal change per infusion rate: the value at the maximum effect from the mean baseline, for each infusion rate tested.

Maximal observed effect per vasopressor: the value of the maximal effect (positive or negative) observed over the whole infusion rate range for a given vasopressor.

For the variables for which dose-response curves were constructed, data were fitted by nonlinear regression analysis using GraphPad Prism^®^ 5.04 and the following parameters was derived:

E_max_ (efficacy): the maximal possible effect, which was determined from the averaging process over 30 s at every 3-minute interval.

ED_50_ (potency): the dose (i.e., infusion rate) inducing 50% of the E_max_.

For each vasopressor, an unpaired Student’s *t*-test with Welch’s correction was performed to compare maximal observed effect values with baseline values and to compare maximal observed values between A and NA animals. This test was also applied for each hemodynamic variable to compare the maximal observed effect induced by selepressin and AVP. For each vasopressor, ED_50_ values in their logarithmic form were compared between A and NA animals using the extra-sum-of-squares F test. A value of p<0.05 was considered as significant.

## Results

### Atherosclerosis Model

**Fig 1:**
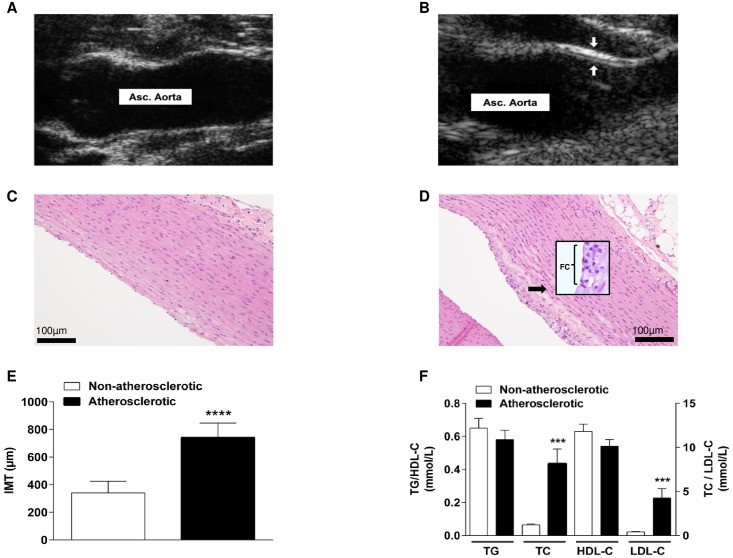
Indicators for atherosclerotic development in the rabbit model. Bi-dimensional mode echographic view of the ascending aorta (Asc. Aorta) in (A) a non-atherosclerotic (non-cholesterol-fed) animal vs. in (B) an atherosclerotic (cholesterol-fed animal) showing an atheromatous plaque (between the arrows) and increased intima-media thickness (IMT). (C) Morphology of the aorta in non-atherosclerotic vs. (D) atherosclerotic rabbit with infiltration of the intima by foam cells (FC). (E) Echographic measurements of the aortic IMT in the non-atherosclerotic vs. atherosclerotic animals, mean ± standard deviation (both n = 15), analyzed using unpaired *t*-test, ****p<0.0001. (F) Serum lipids (TG: triglycerides, TC: total cholesterol, HDL-C: high-density lipoprotein-cholesterol, LDL-C: low-density lipoprotein-cholesterol) on completion of 6 weeks of feeding with normal (non-atherosclerotic) vs. 1% cholesterol-containing diet (atherosclerotic), mean ± standard deviation (both n = 15); analyzed using unpaired *t*-test, ***p<0.001.

Echography demonstrated that in contrast to NA animals (Fig 1A), animals fed with a cholesterol-enriched diet for 6 weeks to induce atherosclerosis (A animals) displayed atheromatous plaques that were present at the level of the ascending aorta (Fig 1B). Histological examination of the aorta showed infiltration of the aortic intima by foam cells in 4 out of the 15 A animals. Foam cell presence was associated with cellular fibrous tissue, whereas none of the NA animals displayed this histological feature (Fig 1C and 1D). One cholesterol-fed animal also presented foam cell presence into the pulmonary artery trunk. Aortic foam cells were also associated with slight aortic mineralization in one A animal and moderate thrombosis in a second animal. The aortic IMT of all the A animals was greater than of the NA animals, which was significant (p<0.0001; Fig 1E). There was a significant increase in total serum cholesterol and low-density lipoprotein-C in the A animals (p<0.001; Fig 1F). The cholesterol diet did not significantly affect TG or high-density lipoprotein-C (Fig 1F).

### Cardiovascular Variables

#### Arterial pressure

**Fig 2:**
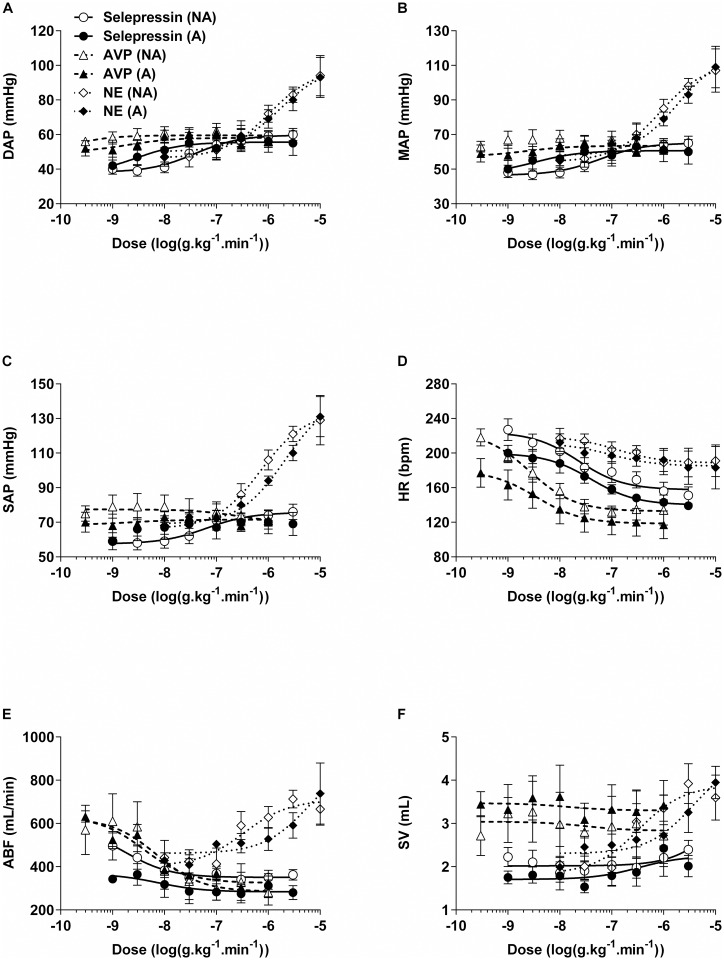
Dose-response curves for hemodynamic variables in non-atherosclerotic (NA) and atherosclerotic (A) rabbits. Selepressin, arginine vasopressin (AVP), or norepinephrine (NE) were administered with incremental infusion rates. A: Diastolic arterial pressure (DAP); B: mean arterial pressure (MAP); C: systolic arterial pressure (SAP); D: heart rate (HR); E: aortic blood flow (ABF); F: stroke volume (SV). A−D: n = 4−5/dose for A and n = 5/dose for NA; E−F: n = 2−5/dose for A and n = 4−5/dose for NA. Curves could not be statistically fitted for AVP effect on MAP in NA rabbits and for selepressin effect on SAP in A rabbits.

**Fig 3:**
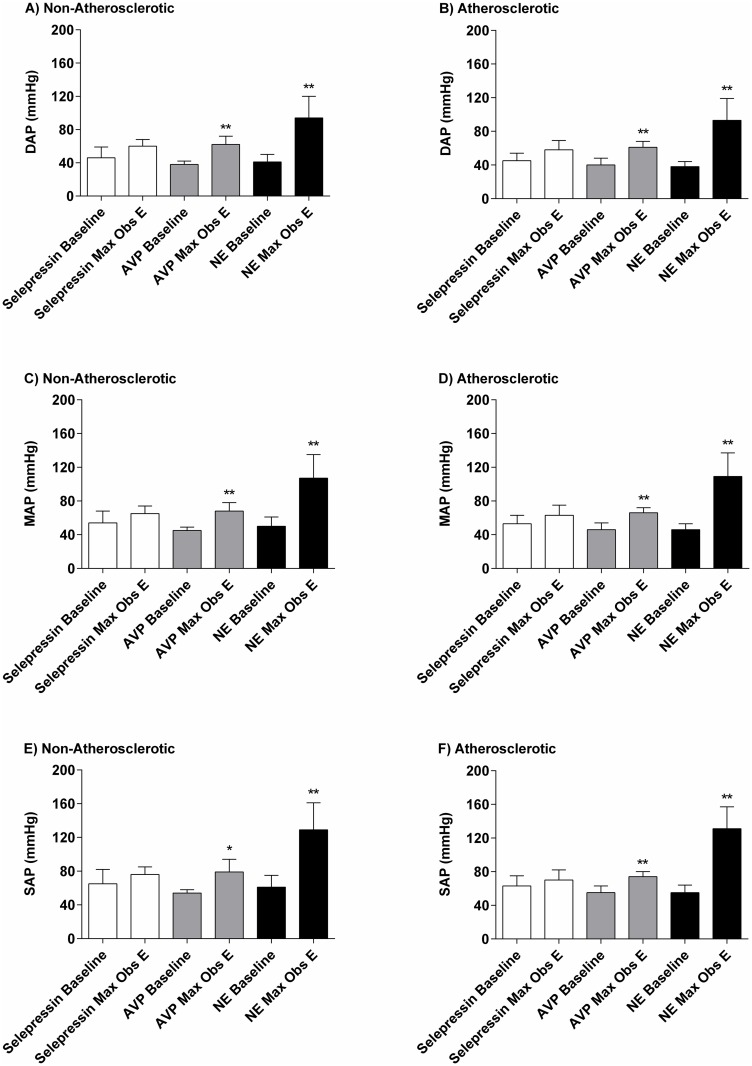
Baseline and maximal observed effect (Max Obs E) for arterial pressure in non-atherosclerotic and atherosclerotic rabbits. Selepressin, arginine vasopressin (AVP), or norepinephrine (NE) were administered with incremental infusion rates. (A and B): Diastolic arterial pressure (DAP); (C and D): mean arterial pressure (MAP); (E and F): systolic arterial pressure (SAP). Difference vs. baseline (n = 5/vasopressor): *p<0.05; **p<0.01.

Baseline diastolic AP, mean AP (MAP), and systolic AP were not significantly different between A and NA animals: 41±8 mmHg vs. 42±9 mmHg, 48±8 mmHg vs. 49±10 mmHg, and 58±10 mmHg vs. 60±13 mmHg, respectively (Fig 2A–2C). Incremental infusion rates of selepressin induced a slight increase in diastolic AP, MAP, and systolic AP, with an increase in MAP in the A and NA animals of up to 10 mmHg and 11 mmHg above baseline, respectively (Fig 2A–2C). There were no significant differences between A and NA animals for the AP dose-response curves in terms of maximal observed effect (Fig 3A–3F) or log ED_50_ values (Table 1).

**Table 1:** Potencies (log ED_50_) of selepressin, arginine vasopressin (AVP), and norepinephrine (NE) for hemodynamic parameters in non-atherosclerotic (NA) and atherosclerotic (A) rabbits.

HemodynamicParameter	Log ED_50_ (log(ng.kg^-1^.min^-1^))					
	Selepressin		AVP		NE	
	NA	A	NA	A	NA	A
DAP	−7.4±0.2	−8.5±0.9	−10.0±7.5	−8.7±1.1	−6.0±0.3	−5.8±0.4
MAP	−7.2±0.3	−8.5±1.3	No fit	−8.6±1.4	−6.1±0.3	−5.7±0.3
SAP	−7.2±0.4	No fit	−6.6±1.9	−8.6±3.2	−6.1±0.2	−5.±0.2
HR	−7.6±0.3	−7.4±0.2	−8.6±0.2	−8.5±0.5	−6.9±0.9	−7.0 ±1.2
ABF	−8.7±0.7	−8.1±1.2	−8.1±0.5	−8.4±0.2	−6.5±0.4	−6.2±0.7
SV	−4.9±6.2	−6.5±1.0	−7.5±5.9	−7.9±5.1	−6.5±0.4	−5.4±0.4

All values are mean ± standard error of the mean. Selepressin and AVP were statistically compared for hemodynamic effects by means of comparison of fits (extra-sum-of-squares F test). No fit: dose-response curve could not be statistically fitted. See legend of Fig 2 for the hemodynamic parameter abbreviations.

Incremental AVP infusion rates caused a mild increase in diastolic AP, MAP, and systolic AP, which for MAP in the A and NA animals was up to 20 mmHg and 23 mmHg above baseline, respectively (Fig 2A–2C). There were no significant differences between A and NA animals for the AP dose-response curves in terms of maximal observed effect values (Fig 3A–3F) or log ED_50_ values (Table 1). Furthermore, the AP dose-response curves for selepressin and AVP did not differ significantly from each other in the A or the NA animals in terms of maximal observed effect values.

In contrast, incremental infusion rates of NE caused a marked increase in diastolic AP, MAP, and systolic AP, which for MAP in the A and NA animals was up to 63 mmHg and 57 mmHg above baseline, respectively (Fig 2A–2C). Of note, increased AP was preceded by a slight to moderate decrease during the first minutes following the initiation of administration of the 10 μg.kg^−1^.min^−1^ infusion rate in four out of five A animals and in two out of five NA animals. Nevertheless, there were no significant differences between A and NA animals for the AP dose-response curves in terms of maximal observed effect values (Fig 3A–3F) or log ED_50_ values (Table 1).

#### Heart rate

**Fig 4:**
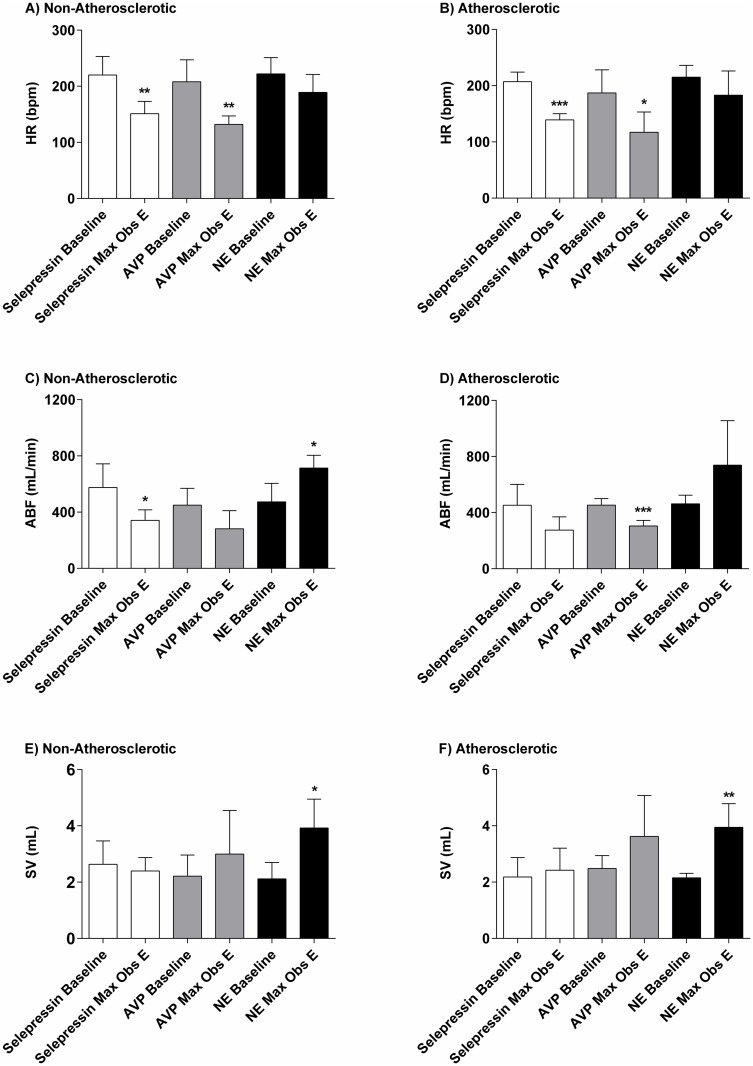
Baseline and maximal observed effect (Max Obs E). Heart rate (HR) (A and B), aortic blood flow (ABF) (C and D), and stroke volume (SV) (E and F) in non-atherosclerotic and atherosclerotic rabbits administered incremental infusion rates of selepressin, arginine vasopressin (AVP), or norepinephrine (NE). Difference vs. baseline (n = 5/vasopressor, except n = 4 for AVP-induced SV Max Obs E): *p<0.05; **p<0.01; ***p<0.001.

Baseline HR values were not significantly different between A and NA animals (203±29 bpm vs. 217±32 bpm) (Fig 4A and 4B). Incremental infusion rates of selepressin caused a moderate decrease in the HR in A and NA animals by up to 68 bpm and 69 bpm below baseline, respectively (Fig 2D). There were no significant differences between A and NA animals for the associated dose-response curves in terms of maximal observed effect values (Fig 4A and 4B) of log ED_50_ values (Table 1).

Incremental infusion rates of AVP induced a moderate decrease in the HR in A and NA animals of up to 70 bpm and 76 bpm below baseline, respectively (Fig 2D). There were no significant differences between the A and NA animals for the associated dose-response curve in terms of maximal observed effect values (Fig 4A and 4B) or log ED_50_ values (Table 1). Furthermore, the HR dose-response curves for selepressin and AVP did not differ significantly from each other in the A or the NA animals in terms of maximal observed effect values.

Incremental infusion rates of NE caused a mild decrease in HR in A and NA animals of up to 32 bpm and 33 bpm below baseline, respectively (Fig 2D). There were no significant differences between the A and NA for the associated dose-response curves in terms of maximal observed effect values (Fig 4A and 4B) or log ED_50_ values (Table 1).

### Biomarkers of Cardiac Injury

**Fig 5:**
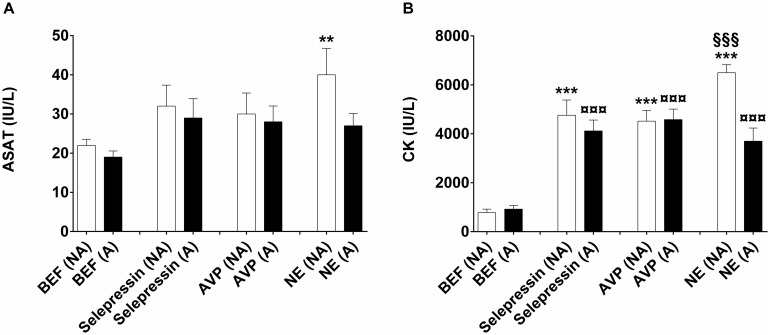
Biomarkers of cardiac injury. (A) Aspartate aminotransferase (ASAT) and (B) creatine kinase (CK) serum levels. Serum levels of ASAT and CK were determined in the anesthetized animal before the onset of treatment (BEF) and just after the last dose level of treatment of selepressin, arginine vasopressin (AVP), or norepinephrine (NE). BEF serum levels were determined after the completion of 6 weeks of feeding with normal (non-atherosclerotic rabbits (NA)) or 1% cholesterol-containing diet (atherosclerotic rabbits (A)). Data was analyzed using one-way analysis of variance followed by Bonferroni’s multiple comparison test (n = 5/vasopressor for A and NA animals). **p<0.01; ***p<0.001: drug (NA) vs. BEF (NA); ^¤¤¤^p< 0.001: drug (A) vs. BEF (A); ^§§§^p<0.001: NE (NA) vs. NE (A).

No electrocardiogram abnormalities (including ST segment elevation) were observed during incremental infusion rates of selepressin or AVP. Some premature ventricular beats were observed during infusion of NE at the highest rate tested (1 μg.kg^−1^.min^−1^), both in the A and NA animals, but was not associated with an increase in the serum concentration of cardiac troponin I from baseline level (<0.16 μg/L; lower limit of quantification). Serum aspartate aminotransferase activity was not significantly different from baseline at the end of administration of selepressin or AVP, both in A and NA animals (Fig 5A). In contrast, it was significantly higher than baseline at the end of NE administration in NA animals but not in A animals (Fig 5A). CK serum concentration was significantly higher than baseline at the end of administration of all three vasopressors both in A and NA animals (Fig 5B)—for NE, it is also noteworthy that CK showed a significantly greater elevation in the NA than in the A animals.

### Aortic Blood Flow

Baseline ABF values were not significantly different between A and NA animals (455±90 mL/min vs. 499±142 mL/min) (Fig 4C and 4D). Incremental infusion rates of selepressin induced a moderate decrease in ABF in A and NA animals of up to 177 mL/min and 234 mL/min below baseline, respectively (Fig 2E). There were no significant differences between A and NA animals for the ABF dose-response curves in terms of maximal observed effect values (Fig 4C and 4D) or log ED_50_ values (Table 1).

Incremental infusion rates of AVP also caused a moderate decrease in ABF in A and NA animals of up to 149 mL/min and 168 mL/min below baseline, respectively (Fig 2E). There were no significant differences between A and NA animals for the ABF dose-response curve in terms of maximal observed effect values (Fig 4C and 4D) or log ED_50_ values (Table 1). Furthermore, the ABF dose-response curves for selepressin and AVP did not differ significantly from each other in the A or the NA animals in terms of maximal observed effect values.

In contrast, incremental infusion rates of NE caused a moderate increase in the ABF rate in the A and NA animals of up to 276 mL/min and 240 mL/min above baseline, respectively (Fig 2E). There were no significant differences between A and NA animals for the ABF dose-response curves in terms of maximal observed effect values (Fig 4C and 4D) or log ED_50_ values (Table 1).

### Stroke Volume

Baseline SV values were not significantly different between A and NA animals (2.27±0.48 mL vs. 2.32±0.71 mL) (Fig 4E and 4F). Incremental infusion rates of selepressin induced minimal changes in SV in A and NA animals (Fig 2F). There were no significant differences between A and NA animals for the SV dose-response curves in terms of maximal observed effect values (Fig 4E and 4F) or log ED_50_ values (Table 1).

Incremental infusion rates of AVP induced a mild non-dose-dependent increase in SV in A and NA animals of up to 1.14 mL and 0.79 mL above baseline, respectively (Fig 2F). There were no significant differences between A and NA animals for the SV dose-response curve in terms of maximal observed effect values (Fig 4E and 4F) or log ED_50_ values (Table 1).

In contrast, incremental infusion rates of NE caused a moderate dose-dependent increase in the SV rate in the A and NA animals of up to 1.80 mL (Fig 2F). There were no significant differences between A and NA animals for the SV dose-response curves in terms of maximal observed effect values (Fig 4E and 4F) or log ED_50_ values (Table 1).

## Discussion

In this study, the cardiovascular safety of the selective V_1A_ receptor agonist selepressin [12–14] and AVP was investigated in comparison with that of the septic shock standard of care NE in a well-established rabbit model of early-stage atherosclerosis. The key observations are that the hemodynamic effects of these three vasopressors, as well as the serum concentrations obtained by incremental infusion rates, did not differ between A and NA animals, suggesting that early-stage atherosclerosis did not influence the pharmacokinetics or cardiovascular pharmacodynamics of these vasopressors. Moreover, selepressin and AVP were not associated with myocardial injury-related electrocardiogram abnormalities, including ST segment elevation, while premature ventricular contractions were observed in some of the NE-treated rabbits (both A and NA) at the highest infusion rate tested.

There was clear evidence of atherosclerosis in the rabbits receiving the cholesterol-enriched diet. Indeed, total serum cholesterol and LDL-C were significantly elevated (Fig 1F) as expected from the literature [23]. In contrast, TG and HDL-C were unaltered. Successful induction of atherosclerosis was confirmed by a greatly increased IMT (Fig 1E), infiltration of the aortic tissue by foam cells (Fig 1D vs. 1C), and clear plaque formation (Fig 1B vs. 1A) [24–27]. Prior to vasopressor administration, atherosclerosis was not associated with any change in AP (Fig 3), as reported previously.^24^

Both selepressin and AVP induced a slight increase in AP (Fig 2A–2C) associated with a moderate decrease in the HR (Fig 2D), possibly of baroreflex origin [28]. The moderate decrease in ABF (Fig 2E) likely reflects a diminished cardiac output caused by the reduced HR (because the SV was maintained—Fig 2F) and possibly also due to increased systemic vascular resistance to both vasopressors [29]. Very similar results were reported in dogs; the selepressin- and AVP-induced increase in AP was also dampened in that animal species by a reduced ABF associated with a diminished HR [19,30]. Because both selepressin and the mixed V_1A_/V_2_ receptor agonist AVP [12,13] demonstrated comparable hemodynamic characteristics, this suggests that the AVP-related cardiovascular effects were triggered mainly by activation of the V_1A_ receptor.

NE, in contrast to selepressin and AVP, induced a marked increase in AP (Fig 2A–2C), which was associated with a lesser decrease in the HR (Figs 2D, 4A and 4B) and a moderate increase in ABF (Figs 2E, 4C and 4D)—the reflection of a dose-dependent increase in SV (Figs 2F, 4E and 4F). These results suggest that an increase in cardiac output mediated by an increase in SV was the main determinant of the increase in AP, consistent with the known positive inotropic effect of NE [31]. NE intravenous infusion bears the risk of cardiovascular liability through premature ventricular contractions, as we observed here in some rabbits at the highest infusion rate tested, which may predispose the heart to ventricular arrhythmia [32]. Moreover, the inotropic effect of NE can greatly increase myocardial oxygen demand, and thus the risk of infarction [33]. Consequently, NE potentially poses a greater cardiovascular risk than selepressin or AVP despite it being used as the standard of care in septic shock.

Interestingly, although the serum concentration of cardiac troponin I—a sensitive and specific biomarker of cardia injury [34]—was not increased by any of the three vasopressors tested, including NE, the serum concentration of CK was significantly increased by all three to the same extent in NA and A animals (Fig 5B). Total CK serum concentration is an insensitive and non-specific biomarker of cardiac injury [34]. Skeletal muscle is by far the tissue possessing the largest amount of CK activity per gram of tissue (5 to 10 times greater than the heart in humans) [35]. Intravenous administration of high doses of vasopressin or epinephrine has been shown to induce rhabdomyolysis accompanied by an increased serum CK concentration [36–39] The magnitude of the increases observed in the present study is similar to that in a rabbit model of surgically-induced acute rhabdomyolysis [40]. Like the serum CK concentration, that of aspartate aminotransferase is an insensitive and non-specific biomarker of cardiac injury [34]. However, we cannot explain the fact that it only significantly increased in NE-treated NA animals (Fig 5A).

The present study had some limitations. Only five animals per group were included for ethical reasons, limiting the power of the study. The study concentrated mainly on hemodynamics, although some histopathological and biomarker aspects of cardiovascular risk were also considered, and only focused on early-stage atherosclerosis. Most importantly, the effect of septic shock on the potential cardiovascular liability of the three vasopressors in this rabbit model of early-stage atherosclerosis was not assessed because anesthesia and sepsis pose a substantial risk for these sensitive animals.

In conclusion, the results from the experimental conditions adopted indicated that there was no relevant additional cardiovascular risk in the atherosclerotic animals following treatment with not only AVP but also with the novel selective V_1A_ receptor agonist selepressin, which is consistent with the early stage of the disease. Further larger animal studies should be performed using models of moderate to advanced atherosclerosis, in particular incorporating septic shock, to assess the safety risk of selepressin and AVP in later stages of the disease. Confirmation of the safety of these vasopressins in such studies in addition to other animal models of heart disease is desirable before considering clinical trials in patients with pre-existing heart disease. The long-term consideration would be the possibility of using new vasopressors such as selepressin in emergency therapeutic intervention, when the pre-existing cardiac health of the patient is still unknown.

## References

[1] Christaki E, Opal SM. Is the mortality rate for septic shock really decreasing? Curr Opin Crit Care. 2008;14: 580–586. 10.1097/MCC.0b013e32830f1e25 18787453

[2] Karlsson S, Varpula M, Ruokonen E, Pettila V, Parviainen I, Ala-Kokko TI, et al Incidence, treatment, and outcome of severe sepsis in ICU-treated adults in Finland: the Finnsepsis study. Intensive Care Med. 2007;33: 435–443. 10.1007/s00134-006-0504-z 17225161

[3] Martin CM, Priestap F, Fisher H, Fowler RA, Heyland DK, Keenan SP, et al A prospective, observational registry of patients with severe sepsis: the Canadian Sepsis Treatment and Response Registry. Crit Care Med. 2009;37: 81–88. 10.1097/CCM.0b013e31819285f0 19050636

[4] Quenot JP, Binquet C, Kara F, Martinet O, Ganster F, Navellou JC, et al The epidemiology of septic shock in French intensive care units: the prospective multicenter cohort EPISS study. Crit Care. 2013;17: R65 10.1186/cc12598 23561510 PMC4056892

[5] Guillamet MCV, Rhee C, Patterson AJ. Cardiovascular management of septic shock in 2012. Curr Infect Dis Rep. 2012;14: 493–502. 10.1007/s11908-012-0279-z 22941043

[6] Agrawal A, Singh VK, Varma A, Sharma R. Therapeutic applications of vasopressin in pediatric patients. Indian Pediatr. 2012;49: 297–305. 22565074 10.1007/s13312-012-0046-0

[7] Collier B, Dossett L, Mann M, Cotton B, Guillamondegui O, Diaz J, et al Vasopressin use is associated with death in acute trauma patients with shock. J Crit Care. 2010;25: 173.e9–e14. 10.1016/j.jcrc.2009.05.003 19682851

[8] Russell JA, Walley KR, Singer J, Gordon AC, Hebert PC, Cooper DJ, et al Vasopressin versus norepinephrine infusion in patients with septic shock. N Engl J Med. 2008;358: 877–887. 10.1056/NEJMoa067373 18305265

[9] Russell JA, Walley KR, Gordon AC, Cooper DJ, Hebert PC, Singer J, et al Interaction of vasopressin infusion, corticoid steroid treatment, and mortality of septic shock. Crit Care Med. 2009;37:811–818. 10.1097/CCM.0b013e3181961ace 19237882

[10] Parillo JE. Septic shock—vasopressin, norepinephrine, and urgency. N Engl J Med. 2008;358: 954–956. 10.1056/NEJMe0800245 18305271

[11] Zaragoza C, Gomez-Guerrero C, Martin-Ventura JL, Blanco-Colio L, Lavin B, Mallavia B, et al Animal models of cardiovascular diseases. J Biomed Biotechnol. 2011;2011: 497841 10.1155/2011/497841 21403831 PMC3042667

[12] Laporte R, Kohan A, Heitzmann J, Wisniewska H, Toy J, La E, et al Pharmacological Characterization of FE 202158, a novel, potent, selective, and short-acting peptidic vasopressin V_1a_ receptor full agonist for the treatment of vasodilatory hypotension. J Pharmacol Exp Ther. 2011;337: 786–796. 10.1124/jpet.111.178848 21411496

[13] Wisniewski K, Galyean R, Tariga H, Alagarsamy S, Croston G, Heitzmann J, et al New, potent, selective, and short-acting peptidic V_1a_ receptor agonists. J Med Chem. 2011;54: 4388–4398. 10.1021/jm200278m 21688787

[14] Rehberg S, Enkhbaatar P, Rehberg J, La E, Ferdyan N, Qi S, et al Unlike arginine vasopressin, the selective V1a receptor agonist FE 202158 does not cause procoagulant effects by releasing von Willebrand factor. Crit Care Med. 2012;40: 1957–1960. 10.1097/CCM.0b013e31824e0fe5 22488005 PMC7461604

[15] Kampmeier TG, Rehberg S, Westphal M, Lange M. Vasopressin in sepsis and shock. Minerva Anestesiol. 2010;76: 844–850. 20935620

[16] Kaufmann JE, Vischer UM. Cellular mechanisms of the hemostatic effects of desmopressin (DDAVP). J Thromb Haemost. 2003;1: 682–689. 12871401 10.1046/j.1538-7836.2003.00190.x

[17] Nussey SS, Bevan DH, Ang VT, Jenkins JS. Effects of arginine vasopressin (AVP) infusions on circulating concentrations of platelet AVP, factor VIII: C and von Willebrand factor. Thromb Haemost. 1986;55: 34–36. 3085262

[18] Spronk PE, Zandstra DF, Ince C. Bench-to-bedside: sepsis is a disease of the microcirculation. Crit Care. 2004;8: 462–468. 10.1186/cc2894 15566617 PMC1065042

[19] Boucheix OB, Milano SP, Henriksson M, Reinheimer TM. Selepressin, a new V_1A_ receptor agonist: hemodynamic comparison to vasopressin in dogs. Shock. 2013;39: 533–538. 10.1097/SHK.0b013e31828aac4b 23429645

[20] Laporte R, Russell J, Landry D, Riviere P. Selective V1a receptor agonist FE 202158 reverses platelet-activating factor-induced hypertension, vascular leak, impaired tissue perfusion, and mortality in rats. Crit Care. 2008;12(Suppl. 2): P407.

[21] Su F, He X, Taccone FS, Xie K, Mouhamed M, Kjølbye AL, et al Early administration of the selective V_1a_ receptor agonist selepressin is superior to arginine vasopressin or norepinephrine in a sheep model of septic shock. Critical Care Med. 2012;40: 123.

[22] Maybauer MO, Maybauer DM, Enkhbaatar P, Laporte R, Wiśniewska H, Traber LD, et al The selective vasopressin type 1a receptor agonist selepressin (FE 202158) blocks vascular leak in ovine severe sepsis. Crit Care Med. 2014;42: e525–e533. 10.1097/CCM.0000000000000300 24674922 PMC4346299

[23] Elshourbagy NA, Meyers HV, Abdel-Melgiud SS. Cholesterol: the good the bad, and the ugly—therapeutic targets for the treatment of dyslipidemia. Med Princ Pract. 2014;23: 99–111. 24334831 10.1159/000356856 PMC5586853

[24] Wilfert K, Drischel K, Unbehaun A, Guski H, Persson PB, Stauss HM. Vascular response to angiotensin II in atherosclerosis: role of the baroreflex. Hypertension. 2000;35: 685–690. 10679518 10.1161/01.hyp.35.2.685

[25] Wetterholm R, Caidahl K, Volkmann R, Brandt-Eliasson U, Fritsche-Danielson R, Gan LM. Imaging of atherosclerosis in WHHL rabbits using high-resolution ultrasound. Ultrasound Med Biol. 2007;33: 720–726. 10.1016/j.ultrasmedbio.2006.11.012 17383806

[26] Gavel NT, Edel AL, Bassett CMC, Weber A-M, Merchant M, Rodriguez-Leyva D, et al The effect of dietary hempseed on atherogenesis and contractile function in aortae from hypercholesterolemic rabbits. Acta Physiol Hung. 2011;98: 273–283. 10.1556/APhysiol.98.2011.3.4 21893466

[27] Kim EJ, Kim BH, Seo HS, Lee YJ, Kim HH, Son HH, et al Cholesterol-induced non-alcoholic fatty liver disease and atherosclerosis aggravated by systemic inflammation. PLoS One. 2014;9: e97841 10.1371/journal.pone.0097841 24901254 PMC4046981

[28] Hasser EM, Bishop VS, Hay M. Interactions between vasopressin and baroreflex control of the sympathetic nervous system. Clin Exp Pharmacol Physiol. 1997;24: 102–108. 9043814 10.1111/j.1440-1681.1997.tb01791.x

[29] Cowley AW Jr. Vasopressin and cardiovascular regulation. Int Rev Physiol. 1982;26: 189–242. 7107151

[30] Cheng CP, Igarashi Y, Klopfenstein HS, Applegate RJ, Shihabi Z, Little WC. Effect of vasopressin on left ventricular performance. Am J Physiol. 1993;264: H53–H60. 8430861 10.1152/ajpheart.1993.264.1.H53

[31] Notterman DA. Inotropic agents—catecholamines, digoxin, amrinone. Crit Care Clin. 1991;7: 583–613. 1863883

[32] Tisdale JE, Patel RV, Webb CR, Borzak S, Zarowitz BJ. Proarrhythmic effects of intravenous vasopressors. Ann Pharmacother. 1995;29: 269–281. 7606074 10.1177/106002809502900309

[33] Lesch M. Inotropic agents and infarct size—theoretical and practical considerations. Am J Cardiol. 1976;37:508–513. 1258787 10.1016/0002-9149(76)90389-1

[34] Lewandrowski KB. Cardiac markers of myocardial necrosis—a history and discussion of milestones and emerging new trends. Clin Lab Med. 2014;34: 31–41. 10.1016/j.cll.2013.11.001 24507785

[35] Jockers-Wretou E, Pfleiderer G. Quantitation of creatine kinase isoenzymes in human tissues and sera by an immunological method. Clin Chim Acta. 1975;58: 223–232. 803419 10.1016/0009-8981(75)90441-6

[36] Moreno-Sanchez D, Casis B, Martin A, Ortiz P, Castellano G, Munoz MT, et al Rhabdomyolysis and cutaneous necrosis following intravenous vasopressin infusion. Gastroenterology. 1991;101: 529–532. 2065928 10.1016/0016-5085(91)90034-i

[37] Pierce ST, Nickl N. Rhabdomyolysis associated with the use of intravenous vasopressin. Am J Gastroenterol. 1993;88: 424–427. 8438851

[38] Eisenburger P, Laggner AN, Lenz K, Druml W. Acute renal failure and rhabdomyolysis after inadvertent intra­arterial infusion of excessive doses of epinephrine during cardiopulmonary resuscitation. Wien Klin Wochenschr. 2000;112: 174–176. 10726331

[39] Fang W, Chen JV, Fang Y, Huang JL. Epinephrine overdose-associated hypokalemia and rhabdomyolysis in a newborn. Pharmacotherapy. 2005;25: 1266–1270. 10.1592/phco.2005.25.9.1266 16164400

[40] Daly KA, Wolf M, Johnson SA, Badylak SF. A rabbit model of peripheral compartment syndrome with associated rhabdomyolysis and a regenerative medicine approach for treatment. Tissue Eng Part C Methods 2011;17: 631–640. 10.1089/ten.tec.2010.0699 21361746

